# Targeting MDM2 by the small molecule RITA: towards the development of new multi-target drugs against cancer

**DOI:** 10.1186/1742-4682-2-38

**Published:** 2005-09-20

**Authors:** L Michel Espinoza-Fonseca

**Affiliations:** 1 Department of Biochemistry, Molecular Biology, and Biophysics, University of Minnesota, Minneapolis 55455, MN, USA

**Keywords:** multi-target drugs, RITA, cancer treatment, blind docking, MDM2, p53 tumor suppressor

## Abstract

**Background:**

The use of low-molecular-weight, non-peptidic molecules that disrupt the interaction between the p53 tumor suppressor and its negative regulator MDM2 has provided a promising alternative for the treatment of different types of cancer. Among these compounds, RITA (reactivation of p53 and induction of tumor cell apoptosis) has been shown to be effective in the selective induction of apoptosis, and this effect is due to its binding to the p53 tumor suppressor. Since biological systems are highly dynamic and MDM2 may bind to different regions of p53, new alternatives should be explored. On this basis, the computational "blind docking" approach was employed in this study to see whether RITA would bind to MDM2.

**Results:**

It was observed that RITA binds to the MDM2 p53 transactivation domain-binding cleft. Thus, RITA can be used as a lead compound for designing improved "multi-target" drugs. This novel strategy could provide enormous benefits to enable effective anti-cancer strategies.

**Conclusion:**

This study has demonstrated that a single molecule can target at least two different proteins related to the same disease.

## Background

The p53 tumor suppressor is one of the principal mediators of cell-cycle arrest and the activation of apoptosis in response to a broad array of cellular injuries [1-4]. In normal unstressed cells, p53 is regulated by a feedback loop with the negative regulator protein MDM2 (murine double-minute clone 2, referred to as human double-minute clone 2, HDM2, in humans) [1,2,5]. A well-known mechanism for the loss of wild-type p53 activity in cancer cells is the overexpression of MDM2, which leads to constitutive inhibition of p53 and thus allows the tumor cells to escape from p53-induced apoptosis [6].

**Figure 1:**
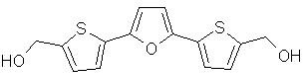
Chemical structure of RITA [2,5-bis(5-hydroxymethyl-2-thienyl)furan].

Recent studies have shown that rescue of p53 function by disruption of the p53-MDM2 interaction may be a promising strategy for developing new anti-cancer drugs [7-9]. To date, different research groups have reported diverse peptidic and non-peptidic molecules that bind at the MDM2-p53 transactivation domain-binding cleft [10-16]. In all cases, these molecules bind to MDM2 and block the p53-MDM2 interaction. In contrast, Issaeva *et al*. reported the small molecule RITA (reactivation of p53 and induction of tumor cell apoptosis, Figure 1), which binds to p53 and targets it for proteasomal degradation [17]. The most interesting feature of RITA was its ability to increase the p53-dependent antitumor effect *in vivo *by inducing a conformational change in p53, which prevented MDM2 binding. In principle, targeting MDM2 or p53 should be sufficient to induce apoptosis effectively in cancer cells. However, considering that biological systems are not static, and that proteins present a certain degree of plasticity due to the pre-existence of conformational populations, the traditional single-drug-single-target approach should be replaced by the single-drug-multiple-target approach. By employing the latter, we can obtain benefits from the "promiscuous" behavior of a potential drug by targeting different proteins with a single molecule [18]. Thus, the possibility that RITA binds to both p53 and MDM2 makes it an attractive lead compound for further development of potent and effective anti-cancer drugs.

In the present study the computational "blind docking" approach [19] is used in order to determine the possibility of RITA binding and its preferential binding sites. It was found not only that RITA can bind efficiently to the MDM2 p53 transactivation domain-binding cleft, but also that is highly specific for its binding site. The results of this study support the effectiveness of the "multi-target" approach in anti-cancer drug design.

## Results and discussion

The objective of this study was to demonstrate that RITA, a drug originally found to bind the p53 tumor suppressor, is also able to bind at the MDM2-p53 transactivation domain-binding cleft, which increases its effectiveness and makes it a lead compound for further anti-cancer drug design efforts.

**Figure 2:**
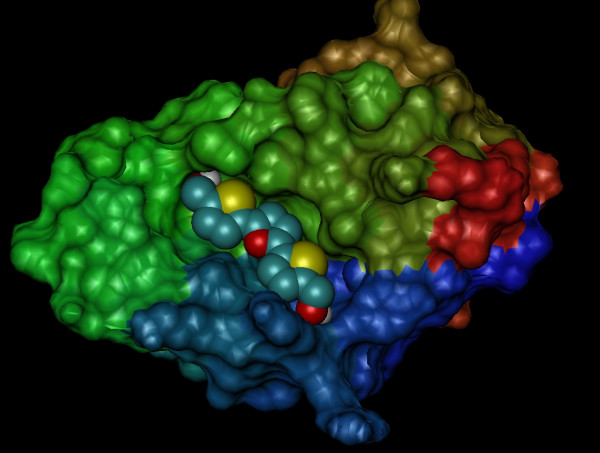
Orientation of the best ranked cluster obtained by using the "blind docking" procedure. RITA is rendered as van der Waals spheres and MDM2 as a surface.

By using the "blind docking" approach, it was found that RITA preferentially binds to the hydrophobic MDM2 p53 transactivation domain-binding cleft. RITA could also bind to other faces of the protein, but this occurred with low frequency. In this case, 81 independent runs out of 100 placed RITA in the MDM2 p53 transactivation domain-binding cleft. The orientation with the most populated cluster is shown in Figure 2. Moreover, "fine docking" focused on the binding cleft showed that 93 out of 100 independent runs accommodated RITA in the same orientation as that observed in the most populated cluster obtained through the "blind docking" procedure. These results imply that RITA is highly specific for the MDM2-p53 transactivation domain-binding cleft. It is also noticeable that RITA covers most of the cleft surface, accommodating horizontally to the cavity and then behaving as a "cap", avoiding p53 to bind to MDM2.

**Figure 3:**
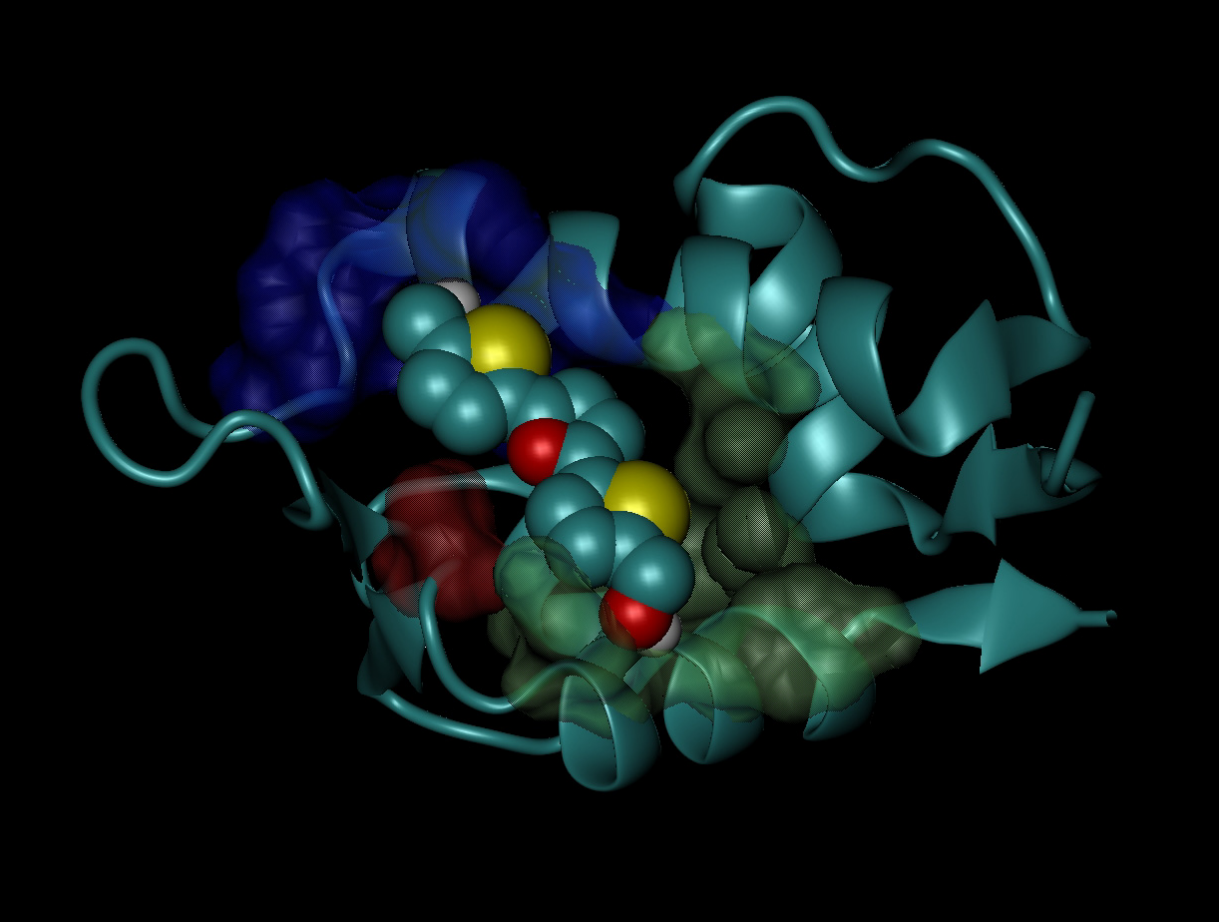
Perspective of the best raked cluster obtained through the "blind docking" procedure. RITA is shown as van der Waals spheres; residues G58, I61, M62 and Y67 are shown as blue surface; residues L54, H96, I99, Y100 and I103 are shown as lime surface; V93 is shown as red surface.

As observed in Figure 3, RITA interacts with the MDM2 as follows: one of the hydroxymethyl-thiophene moieties in the molecule makes contact with residues G58, I61, M62 and Y67, while the other interacts with residues L54, H96, I99, Y100 and I103. Finally, the furan ring makes the only contact with V93. Most of the interactions are favored by van der Waals and hydrophobic interactions. This finding is consistent with the structural composition of both the MDM2-p53 transactivation domain-binding cleft and the thiophene and furan rings, which present large hydrophobic regions.

The calculated binding free energy shows a moderate affinity for MDM2 (Table 1). The stabilizing energy of the complex comes principally from the van der Waals and hydrophobic terms. Subsequently, some hydrogen bonds can be formed between the hydroxyl moieties of the RITA backbone nitrogen, oxygen and hydrogen atoms. However, it is not possible at this stage to determine which residues play an important role in forming such interactions, due to the lack of flexibility of MDM2 during the docking experiments.

**Table 1:** Calculated free energy of binding of RITA obtained through the blind and fine docking experiments. K_d _is the computed dissociation constant, f_occ _is the number of results in the top clusters; N_tot _is the number of clusters generated by AutoDock.

Type of docking	ΔG_b _(kcal/mol)	K_d _(μmol)	f_occ_	N_tot_
Blind	-6.36	22	50	9
Fine	-6.32	23.3	93	3

As mentioned in the introduction, RITA was previously found to bind to the p53 tumor suppressor, which induces its accumulation in tumor cells and leads to selective apoptosis [17]. It was hypothesized that RITA behaved as an allosteric modulator, inducing conformational changes in p53 and preventing it from binding MDM2 but preserving its functional role. Unfortunately, the full structure of the p53 tumor suppressor is not available, which impedes global screening of the best RITA binding site. Nevertheless, there is enough experimental evidence showing that RITA binds to p53. This work demonstrates that RITA could also bind to the MDM2-p53 transactivation domain-binding cleft.

An important point to consider is that it was shown experimentally that RITA does not bind to MDM2 [17]. Nevertheless it was found in this study that the base structure of the compound (the 2,5-di-thiophen-2-yl-furan moiety) was able to bind to the p53-binding cleft. Preliminary docking studies on different NMR structures of MDM2 showed that RITA can actually bind to the same binding cleft over different MDM2 conformations. This suggests that RITA itself might not be a potent MDM2 inhibitor, but the binding affinity for both MDM2 and p53 might be improved by modifying its structure. The apparent inability of RITA to bind to MDM2 in experiments might be due to the need for high concentrations of this compound to attain effective inhibition. This possibility correlates well with our docking simulations, in which the computed dissociation constant of RITA binding to MDM2 is relatively high.

The experimental K_d _obtained for RITA binding to p53 is 1.5 nM, while the computed K_d _of RITA binding to MDM2 is 22 μM. This means that RITA binds some ~15000 times more tightly to p53 than to MDM2. Thus, to use RITA as is would be problematic in principle, since much higher concentrations would be necessary to target both p53 and MDM2 efficiently. The physiological consequences are obvious. On this basis, it is proposed in this study that RITA could be markedly improved to increase its effectiveness on MDM2, while retaining its effectiveness on p53. Thus, structural modifications in RITA would alleviate the lack of selectivity for MDM2, making it an effective multi-target drug.

To visualize the approach presented here better, the following factors should be kept in mind: a) biomolecules are not static entities but are constantly involved in dynamic processes; b) MDM2 displays important conformational transitions when binding to different fragments of the N-terminal domain of p53 [20] and can interact with the core domain of the latter [21,22]; c) p53 may mutate in key residues, hindering RITA binding but not altering its physiological role; d) as pointed by Van Regenmortel, the binding site should not be visualized without considering the binding partner [23]; and e) as remarked by Weaver and co-workers, a promiscuous drug candidate is not a collection of different molecules acting in combination on different receptors implicated in the pathogenesis of a disease, but rather a single molecule capable of binding to a range (albeit a limited range) of targets [24]. Thus, improved RITA derivatives could bind not only to p53 but also to MDM2, which would increase or reinforce its therapeutic effect. In other words, the "multi-target" behavior observed by these compounds would compensate their therapeutic deficiencies because of the dynamic nature of the targets involved and the observed promiscuity of MDM2 over p53. Thereby, RITA could serve as a lead compound for designing improved, low-toxicity and highly effective apoptosis inducers via p53 activation and facilitating the effective inhibition of p53 ubiquitination by MDM2. Its effectiveness would then be improved by its action on multiple pathways related to the disease [18].

Currently, additional simulations are being carried out that allow MDM2 to flex, in order to sample the conformational space more thoroughly, and modifications of the RITA structure are in course. These simulations will help to determine the structural keys involved in the molecular recognition mechanism, to modify the structure of RITA and to improve the activity of a new family of potent anti-cancer drugs.

## Conclusion

The aim of the present study was to support the viability of the multi-target approach in the design of anti-cancer drugs. For this purpose, a recently described system, the MDM2-p53 complex, was successfully used as a study model.

"Blind" and "fine" docking simulations using the AutoDock program revealed that RITA, a molecule originally investigated for binding to the p53 tumor suppressor, can also bind to the MDM2 p53 transactivation domain-binding cleft. As a multi-target drug acting on several proteins related to the same disease, RITA could be more therapeutically effective in the treatment of some types of cancer. These findings open a new and exciting perspective for effective cancer treatment with low-molecular-weight, non-peptidic molecules.

Structural modifications of RITA may help not only to increase the effectiveness of its binding to MDM2 and p53, but also to elucidate the common structural features of p53 and MDM2 in order to improve the anti-cancer activity of a new family of RITA-derived drugs.

## Materials and methods

### Protein preparation

The X-ray structure of human MDM2 in complex with the p53 transactivation domain was used in the present study (PDB code: 1YCR). For docking purposes, the p53 fragment was removed from the original PDB file. Hydrogen atoms were added to the protein and the structure was minimized by 500 steps using the conjugate gradient protocol and employing the CHARMM27 force field implemented in NAMD 2.5 software [25]. Subsequently, non-polar hydrogens were merged from the protein and Kollman united atom charges were assigned. Finally, the protein was equipped with fragmental volumes and solvation parameters.

### Ligand setup

The structure of RITA [2,5-bis(5-hydroxymethyl-2-thienyl)furan] (Figure 1) was optimized at the AM1 semiempirical level, and the Gasteiger-Marsili formalism [20] was employed to derive the partial charges on the atoms. The AUTOTORS utility, included in the latest version of AutoDock (3.0.5) [26], was used to define the torsions of RITA.

### Molecular Docking

Docking simulations were carried out in two stages using version 3.0.5 of the AutoDock program [27]. This program is one of the most reliable docking tools available today because it uses the efficient Lamarckian Genetic algorithm and its scoring function comprises several terms (van der Waals, coulomb potential electrostatics, directional hydrogen bonding, a volume-based solvation term and an estimation of the entropic cost of binding through a weighted sum or torsional degrees of freedom). In addition, the possible binding site need not be specified since the algorithm allows the entire surface of the target to be searched efficiently.

The grid maps representing the protein were calculated with the aid of AutoGrid, a utility of the AutoDock software. Two different grid maps with different dimensions were calculated for the protein. In the first stage, a cubic box of 120 × 120 × 120 points, with a spacing of 0.35 Å between the grid points and centered on the geometric center of the protein, was calculated in order to carry out the "blind docking" experiment. The dimensions of the box were sufficient to cover the entire surface of MDM2. In the second stage, a smaller box (50 × 50 × 50 points, spacing 0.35 Å) was built centered on the most populated binding site, using its geometric center as a reference. This smaller box was employed for performing "fine docking" on the most populated binding site found during the "blind docking" stage. In both cases, docking simulations were carried out using the Lamarckian Genetic Algorithm with an initial population of 300 individuals, a maximum number of 50,000,000 energy evaluations, a maximum number of 50,000 generations, a translation step of 2 Å, a quarternion step of 50° and a torsion step of 50°. For each local search, the pseudo-Solis and Wets algorithm was applied using a maximum number of 300 iterations. Both the blind and refined docking simulations consisted of 100 independent runs. Resulting orientations lying within 2.0 Å in the RMSD were clustered together and represented by the orientation with the most favorable free energy of binding.
